# Medical Secrecy

**Published:** 1921-06-25

**Authors:** 


### THE PUBLIC WELL-BEING.

### Medical Secrecy.

Every now and then something occurs which gives a sudden spurt to the discussion in the daily Press of the inviolability of the confidences between a patient and his doctor. There was such an occurrence a few days ago, when there was again a definite ruling in the Divorce Court that medical men are obliged by law to state in evidence details learned when treating their patients. We note that newspapers for the most part take the view that in this respect the law is unsatisfactory, both in logic and in the public interest. Lawyers are exempt from the necessity of "giving away" statements made to them by their clients, and priests are not compelled (in practice at any rate) to disclose in a court of law the secrets revealed to them in the confessional. The medical profession, it is understood, intend to press for measures to be taken by Parliament to put them in the same position as members of the legal profession. The position for doctors has always been both difficult and delicate. In the recent development of public health policy the question has become much more acute, for it affects in a far more direct way than was the case a few years ago the interests of the profession and a much larger section of the public. In the first place, one of the practical objections to the new medical card insurance system established by the Ministry of Health was the unwonted power it placed in the hands of the panel doctor to reveal the confidences of his numerous patients, or, rather, the impossibility on the part of the doctor—in the present state of the law—to guarantee that he would not use that power if called upon to do so in a court of law. Insurance patients in general probably do not concern themselves at present about the seemingly remote contingency of having the knowledge which they privately convey to the doctor publicly disclosed. But the question would at once become acute if a number of such cases were to occur simultaneously or in quick succession, and the danger then would be that panel patients would instinctively refrain from disclosing to their doctor any information which they might think it indiscreet to part with, but which might (and probably would) have a vital bearing on their condition. Moreover, a real effort (confused though it may be) is now being made to combat systematically the ravages of venereal disease. The victims of that disease, and those who have run the risk of infection, or who have reason to think that they are infected, are being warned and advised that their one hope of cure is to seek promptly medical treatment. There is good reason to believe that this advice is beginning to sink into the public mind. In the past the sense of shame and the fear of possible exposure have undoubtedly been among the main difficulties that the medical profession has had to contend with in controlling the disease. In most of the pamphlets and posters one sees on this subject it is to be observed that secrecy of treatment is carefully insisted upon. If the impression gets abroad that secrecy is not an absolute certainty, because no doctor has it legally within his power to guarantee it, the anti-venereal campaign may suffer quite a serious setback. There seems, at the present time, very good ground for making all doctors "privileged" specifically by law.

